# Prevalence and factors associated with depression in people living with HIV in sub-Saharan Africa: A systematic review and meta-analysis

**DOI:** 10.1371/journal.pone.0181960

**Published:** 2017-08-04

**Authors:** Charlotte Bernard, François Dabis, Nathalie de Rekeneire

**Affiliations:** 1 INSERM, Centre INSERM U1219-Epidémiologie-Biostatistique, Bordeaux, France; 2 University of Bordeaux, School of Public Health (ISPED), Bordeaux, France; Stellenbosch University, SOUTH AFRICA

## Abstract

Depression, one of the most common psychiatric disorders, is two- to three-times more prevalent in people living with HIV (PLHIV) than in the general population in many settings as shown in western countries but remains neglected in sub-Saharan Africa (SSA). We aimed to summarize the available evidence on the prevalence of depression and associated factors according to the scales used and the treatment status in PLHIV in SSA. The pooled prevalence estimates of depression ranged between 9% and 32% in PLHIV on antiretroviral treatment (ART) and in untreated or mixed (treated/untreated) ones, with a substantial variability according to the measurement scale used and also for a given scale. Low socio-economic conditions in PLHIV on ART, female sex and immunosuppression in mixed/untreated PLHIV were frequently reported as associated factors but with no consensus. As depression could have deleterious consequences on the PLHIV life, it is critical to encourage its screening and management, integrating these dimensions in HIV care throughout SSA.

## Introduction

With the improvement in antiretroviral therapy (ART) and the increasing access and use of HIV medical services over almost two decades, a dramatic positive change has been observed worldwide in the demographics of the people living with HIV (PLHIV) [1]. HIV infection can now be considered as a chronic disease in most settings including in sub-Saharan Africa (SSA) [2]. Among the non-communicable disorders observed in PLHIV on ART, insufficient attention has been paid to mental health issues, particularly in SSA where most PLHIV live and are in care.

Depression is one of the most common psychiatric disorders in PLHIV but is neglected in SSA [3]. A recent editorial in AIDS journal reported evidence on the disability associated with HIV-related depression and insisted on the need to act [3]. A meta-analysis conducted in western countries and including studies that used interviews to diagnose major depressive disorders have reported a prevalence of nearly two times higher in PLHIV than in HIV-uninfected subjects [4]. In USA, with the short form of the Composite International Diagnostic Interview, it was also reported a prevalence of two- to three-times higher than in the general population [5] although direct comparisons are rare. The reasons for this higher risk of depression are numerous, including ART side effects, inflammatory processes [3], stigma/discrimination related to HIV/AIDS and fear of premature death [6].

Depression occurrence in PLHIV leads to alteration of economic productivity, decrease of working abilities, social isolation, physical decline and difficulties in solving problems, again more severely in PLHIV [3]. Depression has been shown to predict non-adherence to ART [7,8]. A recent study reported that non-adherent patients had a 3-fold higher risk of presenting moderate to severe depressive symptoms in comparison to adherent patients [9]. Studies in Africa revealed that in PLHIV, depression is also associated with poorer health status overall, including low weight gain, low CD4 progression [10], suicide [11] but also with faster progression to AIDS and increased mortality [3]. Depression could be clinically quite different from the depression among uninfected patients, as reported in one study, revealing that compared to depressed uninfected subjects, depressed PLHIV have a later onset of depressive illness, are more likely to take medications before the onset of depression and have more severe symptoms (ie more critical of themselves, poorer sleep, tired, loss of appetite…) [6]. In this context, depression could seriously compromise ART outcomes at individual and population levels.

As depression in PLHIV emerges as a public health issue, the risk of burden on the health care systems and human resources is significant, especially in SSA. Two reviews aimed to present the consequences of depression on ART adherence [12,13] and clinical outcomes [13]. They were not centered entirely on depression and combined all age groups including children. Another review focused on the reliability and the validity of the tools to screen for depression [14]. Two of these publications presented pooled prevalence estimates but with publications before 2012 for one of them [12] and without differentiating the scales used for the other one [14]. The aim of this new systematic review and meta-analysis is to summarize the most recent available evidence up to April 2016 in adult PLHIV living in SSA on: 1) the prevalence of Major Depressive Disorders (MDD), (ie patients meeting diagnosis criteria of depression according to the Diagnosis and Statistical Manual of Mental Disorders [DSM]) and depressive symptoms, (ie defined based on a screening instrument with a score higher than a specified threshold), and 2) the risk factors for depression. The strength of this updated review is to report the results according to the measurement scales used and the care and treatment characteristics (treated PLHIV vs untreated or mixed PLHIV, ie samples mixing treated and untreated patients). We combined a meta-analysis and a qualitative review to report thoroughly the data available on the topic. Pooled prevalence estimates were calculated when possible.

## Methods

### Eligibility criteria

The publications were searched on Medline, Scopus, specific neuropsychologic literature databases (PsycInfo, PsycArticles, Psychology and behavioral sciences collection) and specific African databases (African Index Medicus and African journal online) using the following terms (Mesh terms): "HIV Infections" AND ("Depressive Disorder" OR "Depression") AND ("Africa" OR "Africa South of the Sahara"). We also added filters on the date of publication (1996 to April 2016) and the age of the patients (≥18 years old) (S1 File). We included cross-sectional and longitudinal studies assessing the prevalence and the factors associated to major depression and depressive symptoms in adult PLHIV. Four papers were identified by complementary researches and four others were recommended by an expert. No paper corresponding to our criteria was found in the grey literature (http://www.opengrey.eu).

**Fig 1:**
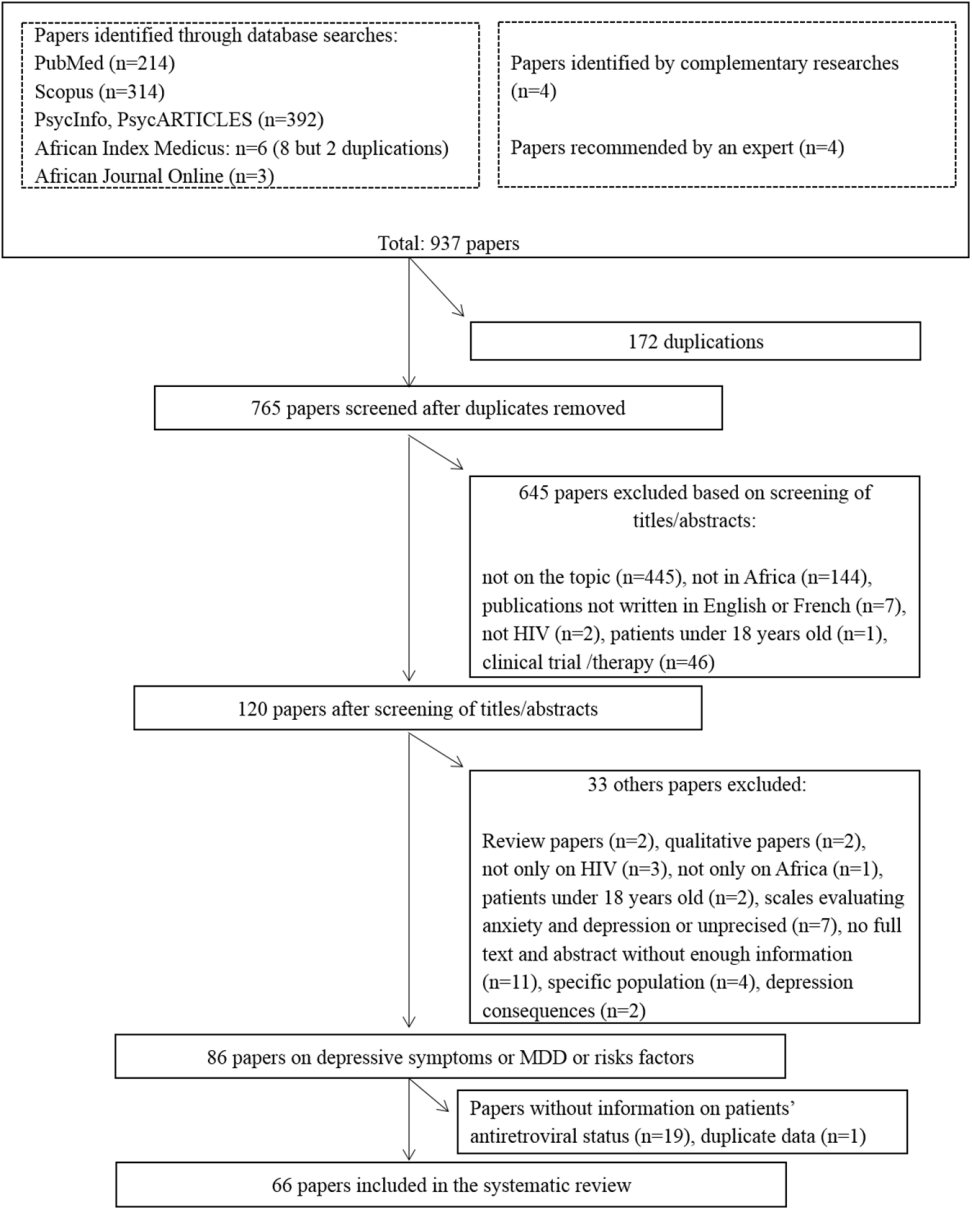
Flow diagram for systematic review process.

After a first screening of titles or abstracts to remove duplicates and exclude papers out of the scope, 120 papers were identified (Fig 1). Among them, we excluded review papers (n = 2), qualitative papers (n = 2), papers neither focusing on PLHIV (n = 3), nor on African patients (n = 1) and those combining adults with patients under 18 years old (n = 2). We also excluded papers using inaccurate measurement scales or scales evaluating both anxiety and depression without giving specific information on depression (n = 7), papers with no full text and abstract with insufficient information (n = 11), papers with very specific populations (n = 4) or those focusing on depression consequences (n = 2). Among the 86 remaining papers, 19 papers were excluded when no information on patients’ ART status was available (authors were contacted by email but no answer was obtained) and one with duplicate data reported.

Altogether, 66 papers were included in the review. Fifty-five full-text papers reported the prevalence of major depression and/or depressive symptoms (10 on MDD only, 40 on depressive symptoms only, and five on both) whereas 11 reported information only on factors associated with depression. Thirteen papers were published before 2010 and 53 (80.3%) between 2010 and April 2016. We reported pooled prevalence estimates for the studies using the scales with the same number of items and the same cut-off, e.g. for the Center for Epidemiologic Studies–Depression (CES-D) Scale [15] 20 items; we did not include the studies using CES-D with 11 or 10 items. Altogether, 25 papers were included to calculate the pooled prevalence of depressive symptoms (14 of them distinguished ART-treated PLHIV); nine papers allowed the computation of the pooled prevalence of MDD (3 of them distinguished ART-treated PLHIV).

### Tools and validity

Among the 16 papers reporting MDD prevalence estimates, the Mini-International Neuropsychiatric Interview (MINI) [16], the usual gold standard, was used in nine of them. The MINI is a brief structured diagnostic interview across the major Axis I psychiatric disorders in the *DSM-III-R*, *DSM-IV*, and the *International Statistical Classification of Diseases and Related Health Problems* (*ICD-10)*.

The majority of the studies selected used screening scales based on DSM-IV depression criteria to evaluate the severity of depressive symptoms. Among the 45 papers evaluating depressive symptoms, 14 (31.1%) used the CES-D scale. The CES-D is a 20-item self-rating scale that assesses symptoms experienced by the participant during the past week, such as depressed mood, feeling guilty, helplessness and somatic symptoms (loss of appetite, sleep disturbance). The total score ranges from 0 to 60 with scores ≥16 representing significant depressive symptoms. Eight reports (17.8%) used the Patient Health Questionnaire (PHQ-9) [17,18]. The PHQ-9 is a 9-item self-rating scale evaluating the key symptoms of depression during the past two weeks. The total score ranges from 0 to 27 with five categories of severity: minimal (0–4), mild (5–9), moderate (10–14), moderately severe (15–19) and severe (20–27). The 15-item depression sub-scale of the Hopkins Symptom Checklist (HSCL-D) [19] was also reported in eight (17.8%) papers, with a threshold >1.75 defining significant depressive symptoms. The Beck Depression Inventory (BDI) [20], a 21-item self-report measure, was reported in six (13.3%) papers.

In SSA studies, with this 16 score cut-off, the CES-D scale was reported having a good sensitivity but a limited specificity (79% to 91% and 44% to 81%, respectively) [21–23]. In a recent meta-analysis, pooled sensitivity and specificity for the CES-D, based on four African studies were high for the threshold ranges between 16 and 22 (sensitivity 82% [95% CI, 73–87], specificity 73% [95% CI, 63–80]).[14] For a PHQ-9 cut-off ≥10, three African studies reported a variable sensitivity but a high specificity (27% to 91.6% and 70.8% to 95.6% respectively) [21,24–26]. No validity study was available in Africa for the HSCL-D scale. There is no consensus on what cut-off to use for the BDI.

### Statistical analyses

For the meta-analysis, forest plots were used to present pooled prevalence and 95% confidence interval (CI) of MDD and depressive symptoms based on the scales used and the care and treatment status (treated PLHIV vs untreated or mixed PLHIV). For each scale, we differentiated the results from the meta-analysis and from the qualitative review, by adding “Meta-analysis” or “Qualitative review” at the beginning of the paragraphs. The weight of each study was taken into account to estimate the pooled prevalence, as indicated on the forest plots. Between-study heterogeneity was assessed using I^2^ statistics [27]. The analyses were performed with MetaXL version 5.1. Finally, the quality of the studies included in the meta-analysis was assessed using the Grading of Recommendations Assessment, Development, and Evaluation—Guideline Development Tool (GRADEpro GDT). GRADEpro GDT (http://www.guidelinedevelopment.org/) is an easy and free tool, available online to facilitate the creation of evidence tables, allow to present clearly the criteria required for the quality assessment of the publications and facilitate the conclusion about the quality of the publications. For the tables, variety of formats are available: we have chosen the GRADE Evidence Profile.

The factors associated to MDD or depressive symptoms are reported based on the results of the meta-analysis.

This review was conducted in accordance with Preferred Reporting Items for Systematic Reviews and Meta-Analyses (PRISMA) guidelines (Table A in S2 File).

## Results

### Prevalence of MDD

Results of studies reporting the prevalence of MDD are shown in Tables 1 and 2 according to the tool used and the treatment status.

**Table 1:** Major depressive disorders in HIV-infected adults evaluated by the Mini International Neuropsychiatric Interview (MINI).

First Author, year by chronological order	Country	Study years	HIV+ N	HIV- N	Demographic data			Clinical data			Tools	Main results or Prevalence (%) (95% CI)
					Age (years)	Men (%)	Education	CD4 cell count /mm^3^	CDC/WHO stage (%)	ART (%)		
Adewuya et al, 2008 [28]	Nigeria	NA	87	NA	31–40 yo: 49.4% >40 yo 13.8%	43.7%	Secondary: 55.2%Tertiary: 9.2%	NA	NA	100%	MINI	28.7% (11.8% major / 15.1% minor)
Nakimuli-Mpungu et al, 2011 [29]	Uganda rural	2011	500	NA	40 (10.7)*	30.2%	Post-Secondary:17.4%	CD4<200: 26%	WHO3/4: 41.8%	100%	MINI	25% (9.5% both current and lifetime, 15.2% only lifetime)
Nakimuli-Mpungu et al, 2013 [30]	Uganda	2011	400	NA	>40 yo: 47%	34%	Post-Primary: 16%	NA	WHO 3/4: 41.7%	100%	MINI	• Current: 12.25% • ART adherence vs non-adherence • Lifetime: 19% vs 28.5% • Current: 8% vs16.5%
Olley et al, 2006 [31]	South Africa	2002–2003	149	NA	30 (7)*	29.5%	NA	346.32 (236.21)*	NA	1.3%	MINI	• At baseline: 34.8% • At follow-up: 20% (new cases 8.1%)
Myer et al, 2008 [22]	South Africa	2004–2005	465	NA	33 (29–38)**	25%	Post-Primary: 16%	234** CD4<200: 37%	NA	48%	MINI	14%
Akena, Musisi et al, 2012 [32]	Uganda	2011	368	NA	38.8 (9.81)*	28%	Secondary / post-secondary: 53.8%	329.7 (186.95)*	WHO 3 / 4: 25.8% / 11.1%	“a number of PLWHA were accessing ART”	MINI	17.4%
Nakku et al, 2013 [33]	Uganda	NA	618	NA	25–34 yo: 38.6% ≥45yo: 16.7%	27.3%	Secondary / post-secondary: 42.7%	CD4<250: 42.7%	NA	64.6%	MINI+	8%
Besa et al, 2015 [34]	Zambia	NA	185	NA	39*	28.1%	Secondary: 42.2%	CD4<350: 32.5%	NA	98.4%	MINI	7%
Spies et al, 2009 [35]	South Africa	2004–2005	429	NA	range 18–65 yo	24%	NA	NA	NA	treatment seeking	MINI	• Current MDD: 53.12% • Past MDD: 28.7%

Abbreviations: ART: Antiretroviral Therapy; CI: Confidence Interval; IQR: InterQuartile Range; NA: Not available; SD: Standard Deviation; yo: years old. **Longitudinal analyses are highlighted in grey.**

*Mean (SD)

** Median (IQR)

NB: Articles are presented in function of ART status of included patients

**Table 2:** Major depressive disorders in HIV-infected adults evaluated by other scales than the MINI.

First Author, year by chronological order	Country	Study years	HIV+ N	HIV-N	Demographic data			Clinical data			Tools	Main results or Prevalence (%) (95% CI)
					Age (years)	Men (%)	Education	CD4 cell count /mm^3^	CDC/WHO stage (%)	ART (%)		
Olisah et al, 2010 [36]	Nigeria	2006	310	NA	35.5 (8.97)*	31.6%	Secondary: 37.4% Tertiary: 27.1%	NA	NA	100%	ICD-10	14.2%
Gaynes et al, 2012 [37]	Cameroon	2010	400	NA	41 (34–47)**	26%	Post-Primary (>6 years): 39%	NA	NA	100%	CIDI	7% in prior year, 5% in prior 6 months, 3% in past month; 21% during lifetime
Ngo et al, 2013 [38]	Uganda	2009	40	NA	<40 yo: 52.5%	32.5%	Secondary / post-secondary: 80%	204 (147)* CD4≤250: 69%	NA	100%	Semi-structured interview Diagnostic made in clinic psychiatric	65% (26/40 patients)
Lawler et al, 2011 [39]	Bostwana	2008	120	NA	37.5 (6.5)*	50%	Secondary: 48% Tertiary 15%	360.4 (181.4)* CD4<200: 20%	NA	97.5%	Mood Module (MM) of the Primary Care Evaluation of Mental Disorders (Prime-MD)	24%
Wagner et al, 2012 [40]	Uganda	2010–2011	602	NA	NA	NA	NA	NA	NA	49.8%	NA	13%
Nyirenda et al, 2013[41]	South Africa rural	2003–2007	422		≥50 yo	NA	NA	NA	NA	yes/no	CIDI	Major: 14.8% (9.9–19.7%)HV+ vs 30.1% (24.0–36.2%) HIV-affected

Abbreviations: ART: Antiretroviral Therapy; CI: Confidence Interval; CIDI: World Health Organization’s Composite International Diagnostic Instrument; ICD-10: International Classification of Diseases; IQR: InterQuartile Range; NA: Not available; SD: Standard Deviation; yo: years old.

*Mean (SD)

** Median (IQR)

NB: Articles are presented in function of ART status of included patients

#### MINI

Results are presented in Table 1.

**Meta-analysis**

**Fig 2:**
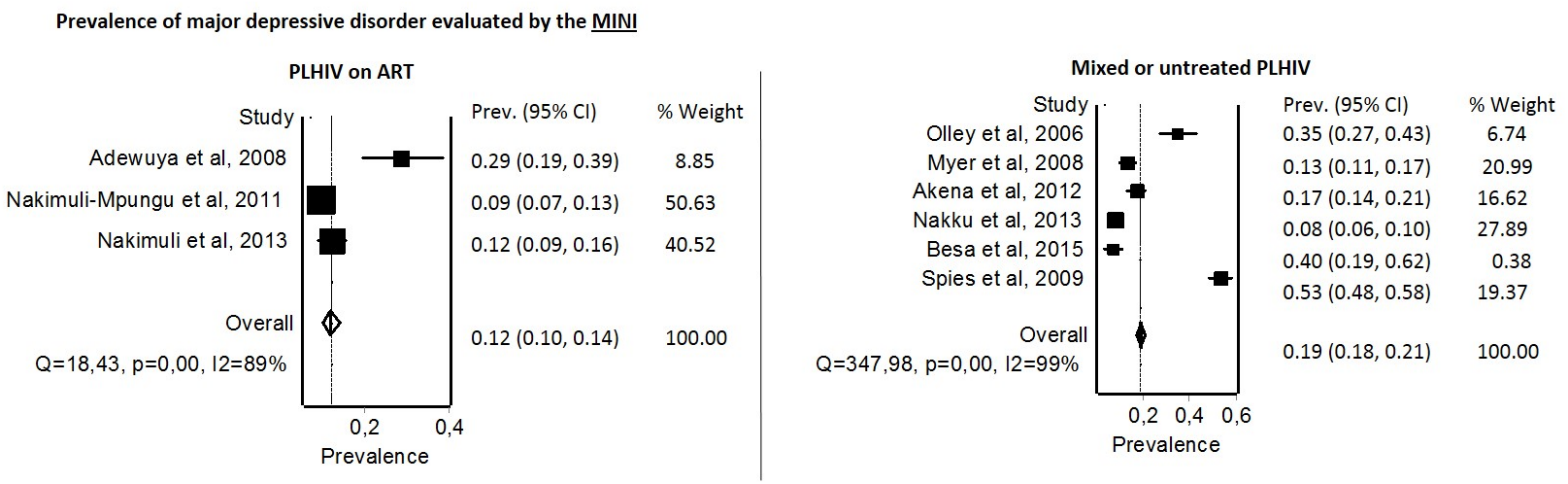
Forest plots presenting the prevalence of MDD according to the tool used and the treatment status. The between-study heterogeneity is reported using I^2^ statistics and its associated p-value. Prev: Prevalence; CI: confidence interval.

In untreated or mixed groups of PLHIV, the pooled prevalence of depressive symptoms was 19% [95% CI: 18–21] with a high between-group heterogeneity (I^2^ = 99%) (Fig 2-right) [22,31–35]. In PLHIV on ART, the pooled prevalence of depressive symptoms was 12% [95% CI: 10–14] with a high between-group heterogeneity (I^2^ = 89%) (Fig 2-left) [28–30].

**Qualitative review**

In untreated or mixed groups of PLHIV, in South Africa, a longitudinal study revealed that MDD was one on the most prevalent psychiatric disorders both at baseline (34.8%) and during follow-up (20%). Although more than half of the depressed PLHIV at baseline no longer met criteria for depression at follow-up times, new cases were diagnosed over time (8.1%) [31].

#### Other tools

Results are presented in Table 2.

**Meta-analysis**

No pooled prevalence was calculated due to the variety of scales used.

**Qualitative review**

In mixed groups of PLHIV, the prevalence of MDD ranged from 13% to 24% [39,40]. The prevalence of MDD was significantly higher in PLHIV aged 50 years old or above (30.1% vs 14.8%) in comparison with HIV-uninfected patients [41].

In PLHIV on ART, the prevalence of MDD ranged from 3% to 14.2% [36,37]. Only one study in Uganda where the diagnosis was made by a psychiatrist reported that 65% of the PLHIV had MDD. Among them, 61.5% were diagnosed after becoming HIV-positive and 38.4% after starting ART [38].

### Prevalence of depressive symptoms

Results of studies reporting the prevalence of depressive symptoms severity are shown in Tables 3–7 according to the tool used and the treatment status.

**Table 3:** Severity of depressive disorders in HIV-infected adults evaluated by the Center for Epidemiologic Studies—Depression Scale (CES-D).

First Author, year by chronological order	Country	Study years	HIV+ N	HIV- N	Demographic data			Clinical data			Tools	Main results or Prevalence (%) (95% CI)
					Age (years)	Men (%)	Education level (%)	CD4 cell count /mm^3^	CDC/WHO stage (%)	ART (%)		
**CES-D 20 items**												
Poupard et al, 2007 [42]	Senegal	NA	200	NA	Women: 44 Men: 36**	36.5%	NA	341** range: 31–953; CD4<300: 54%	NA	100%	• Cut-off ≥17 for men • Cut-off ≥23 for women	• 18% (12–23%) • (19% for PLHIV with protease inhibitor vs 17% for PLHIV with efavirenz)
Nakasujja et al, 2010 [43]	Uganda	2005–2007	102	25	34.2(6.23) vs 30.3(3.99)*	27% vs 60%	9.1 (4.3) vs 10.4 (4.2)*	130 (69.5)*	NA	initiating	Cut-off ≥16	• At baseline: 53.39% (44.1–63.6%) vs 28% (10.4–45.6%); OR 2.86 (1.03–7.95) adjusted on age and gender • At 3-month: 36% vs 13% • At 6-month: 30% vs 24%
Olisah et al, 2010 [36]	Nigeria	2006	310	NA	35.5 (8.97)*	31.6%	Secondary: 37.4% Tertiary: 27.1%	NA	NA	100%	Cut-off ≥16	21.3%
Kitshoff et al, 2012 [44]	South Africa	2010	146	NA	36 (31–42) **	27.4%	Primary: 51% Tertiary and post tertiary: 44%	312 (193–471)**	NA	100%	Cut-off ≥16	62%
Myer et al, 2008 [22]	South Africa	2004–2005	465	NA	33 (29–38)**	25%	Primary: 86%	234**; CD4<200: 37%	NA	48%	Cut-off NA	45%
Farley et al, 2010 [45]	Nigeria	2007	399	NA	≥18yo	31.8%	Post-secondary: 29.8%	CD4<200: 50.1%	NA	55.6%	Cut-off ≥16 or ≥21	• ≥16: 13% • ≥21: 6%
Seth et al, 2014 [46]	Kenya, Namibia, Tanzania	2009–2010	3538	NA	37,2 (8.4)*	41.9%	NA	348 (210–504)**; CD4<200: 23.7%	NA	64%	Mild: cut-off 16–26 Severe: cut-off >27	• 28% (16% mild; 12% severe) • Women 32% (18% mild, 14% severe) • Men 22% (13% mild, 9% severe)
Kaharuza et al, 2006 [47]	Uganda	2003–2004	1017	NA	31-40yo: 44%; >50yo: 9%	23%	Post-primary: 21%	CD4<200: 45%	NA	naïve	• Cut-off ≥23 • Distress (scores 16–22)	47% / distress: 19%
Singh et al, 2008 [23]	South Africa	2007	20	NA	34 (30–39)**	40%	NA	35 (22–91)**	NA	naïve	Cut-off ≥16	40%
**CES-D 11 items**												
Simbayi et al, 2007 [48]	South Africa	NA	1063	NA	≥36yo: 28%	39.5%	NA	NA	NA	50%	Cut-off ≥16	30%
**CES-D 10 items**												
Alemu et al, 2012 [49]	Ethiopia	2010	1722	NA	37.93 (8.97)*	38.7%	NA	119** CD4<200: 28%	NA	100%	10 items	13.1% women / 8.7% for men
Klis et al, 2011 [50]	Gambia	2007	44	NA	32.3**	21.4%	≥1 year: 52.3%	CD4 <200: 30%	NA	0%	• 10 items • Cut-off ≥11	40.9%

Abbreviations: ART: Antiretroviral Therapy; CI: Confidence Interval; IQR: InterQuartile Range; NA: Not available; SD: Standard Deviation. **Longitudinal analyses are highlighted in grey.**

*Mean (SD)

** Median (IQR) NB: Articles are presented in function of ART status of included patients

**Table 4:** Severity of depressive disorders in HIV-infected adults evaluated by the Patient Health Questionnaire (PHQ-9).

First Author, year by chronological order	Country	Study years	HIV+ N	HIV- N		Demographic data			Clinical data			Tools	Main results or Prevalence (%) (95% CI)
						Age (years)	Men (%)	Education level (%)	CD4 cell count /mm^3^	CDC/WHO stage (%)	ART (%)		
Wagner et al, 2011 [51]	Uganda	NA	602		NA	36* Range: 20–62	32%	≤ primary: 54% Secondary: 37%	216*Range 1–846	45%	starting	≥10	13%
Asangbeh et al, 2015	Cameroon	2012	200		NA	43.4(11.5)*	34.7%	Only primary: 71.8%	40.1% CD4>500	NA	100%	≥10	28.7%
Belenky et al, 2014 [52]	Tanzania	2008–2009	403		NA	42 (36–48)**	31%	• Primary:74% • Secondary: 17.4%	304(191–443)**	NA	100%	• Mild/moderate/severe ≥5 • Moderate or severe ≥10	• Mild, moderate or severe: 23% • Moderate or severe: 10%
Endeshaw et al, 2014 [53]	Ethiopia	NA	55		NA	34.68 * (IQR40-28)	36.4%	7^th^-12^th^ grade: 41.8% ≥diploma: 10.9%	336.5*	NA	100%	NA	• Mild: 29.09% • Moderate: 20% • Moderately severe: 10.91%
Musisi et al, 2014 [54]	Uganda	2010–2011	386		NA	35.7(8.7)*	41.7%	Secondary: 16.5%	163(86) *	16.1%	starting	• Minor: 5–9 • Major: >9	• Minor: 19.2% • Major: 10.9%
L’akoa et al, 2013 [55]	Cameroon	2011	100		NA	40.4(10.4)*	48%	University level: 28%	234(129)*; 39% <200	NA	40%	• Moderate 10–14 • Moderately severe 15–19 • Severe ≥20	• 63% (53.2–71.8%) • Moderate: 46% (36.6–55.7%) • Moderately severe: 16% (10.1–24.4%) • Severe: 1% (0.2–5.4%)
Wagner, Ghosh-Dastidar et al, 2014 [56]	Uganda	2008–2011	750		NA	34.5 *	42%	Secondary / post-secondary 16%	222*	NA	67% starting	• Minor 5–9 • Major >9	• Minor : 28% • Major : 6%
Wagner, Ghosh-Dastidar et al, 2014 [57]	Uganda	2008–2011	1731		NA	35.6*	35%	Secondary / post-secondary 15%	209*	NA	69.5% starting	• Minor 5–9 • Major >9	• Minor : 28% • Major : 9%

Abbreviations: ART: Antiretroviral Therapy; CES-D: Center for Epidemiologic Studies—Depression Scale; CI: Confidence Interval; IQR: InterQuartile Range; NA: Not available; SD: Standard Deviation.

*Mean (SD)

** Median (IQR)

NB: Articles are presented in function of ART status of included patients

**Table 5:** Severity of depressive disorders in HIV-infected adults evaluated by the Beck Depression Inventory (BDI).

First Author, year by chronological order	Country	Study years	HIV+ N	HIV- N	Demographic data			Clinical data			Tools	Main results or Prevalence (%) (95% CI)
					Age (years)	Men (%)	Education level (%)	CD4 cell count /mm^3^	CDC/WHO stage (%)	ART (%)		
**BDI 13 items**												
Amberbir et al, 2008 [58]	Ethiopia	2006–2007	400	NA	• 30** • Range • 19–58	40.3%	Secondary 50.3%	CD4<200: 72.2%	• WHO 3/4: 66.3% / 24.8%	100%	Cut-off ≥10	• At baseline: 55.8% • At follow-up: 49.6%
**BDI 21 items**												
Kagee et al, 2013 [59]	South Africa	NA	200	NS	34.05 (7.6)*	21%	• Secondary: 65.3% • Tertiary: 7.1%	NA	NA	100%	• Moderate 17–30 • Severe ≥31	• Moderate 28.5% • Severe 16.2%
Nel et al, 2013 [9]	South Africa	NA	101	NA	35.04 (7.05)*	17.8%	NA	NA	NA	100%	• Mild 14–19 • Moderate 20–28 • Severe 29–63	• Mild 11.7% • Moderate 22.3% • Severe 18.1%
Kagee et al, 2010 [60]	South Africa	NA	85	NS	32.8 (7.6)*	24.7%	• Secondary: 10% • Tertiary: 90%	NA	NA	yes/no	• Mild 10–18 • Moderate 19–29 • Severe 30–63	• Mild 24.7% • Moderate 20% • Severe 17.6%
Lawler et al, 2011 [39]	Bostwana	2008	120	NA	37.5 (6.5)*	50%	• Secondary: 48% • Tertiary: 15%	360.4 (181.4)* CD4<200: 20%	NA	97.5%	• Fast screen 7 items • Cut-off ≥4	38% (mild: 59%; moderate: 27%; severe: 14%)

Abbreviations: ART: Antiretroviral Therapy; CI: Confidence Interval; IQR: InterQuartile Range; NA: Not available; SD: Standard Deviation.

*Mean (SD)

** Median (IQR)

NB: Articles are presented in function of ART status of included patients

**Table 6:** Severity of depressive disorders in HIV-infected adults evaluated by the Hopkins Symptoms Checklist (HSCL).

First Author, year by chronological order	Country	Study years	HIV+ N	HIV- N	Demographic data			Clinical data			Tools	Main results or Prevalence (%) (95% CI)
					Age (years)	Men (%)	Education level (%)	CD4 cell count /mm^3^	CDC/WHO stage (%)	ART (%)		
**HSCL Cut-off>1.75**												
Tsai et al, 2012 [61]	Uganda	since 2005	456	NA	Women: 34 (28–38.4) Men: 38.5 (33–44.9)**	29%	Secondary: Women: 22.2%; Men: 25.8%	• Women: 210.5 (138–304); • Men 177 • (107–265)**	NA	initiating	12 cognitive-affective items(without somatic symptoms)	At baseline: 20.4% for women vs 3.9% for men
Psaros et al, 2015 [62]	Uganda	2005–2010	453	NA	34.9(8.3)*	69.3	≥Secondary: 24.9%	150.2(104.3)*	NA	initiating	25 items	38%
Wroe et al, 2015 [8]	Rwanda	2008	292	NA	38.2 (11.8)*	37%	NA	at ART initiation: 187.5 (95–256.5)**	WHO 3/4: 44.4% / 3.8%	100%	15 items	11.2%
Martinez et al, 2008 [63]	Uganda	2005	421	NA	36 (31–42) **	36.8%	39.4% beyond primary education	218 (91–395) (n = 202)	WHO 3/4: 46.8% / 15.2%	57.1%	11 items; without somatic symptoms	18.8%
Hatcher et al, 2012 [64]	Uganda	2007	270	NA	34 (28–38)**	0%	Secondary : 23%	20 (136–308)**	WHO 4 : 30.4%	44.8%	15 items	23.7%
Chan et al, 2015 [65]	Uganda	2005–2012	534	NA	34.5 (8.9) *	29%	Post- primary: 26.4%	186.8(140.3)*	WHO 3/4: 28.7% / 4.6%	naïve	12 cognitive-affective items(without somatic symptoms)	• At baseline: 28.8% (34.5% for women ; 12.9% for men) • 3.1% of relative decline in the mean depression symptom severity score in each year of study recruitment after the first year
**HSCL Cut-off>2**												
Martin et al, 2014 [66]	Uganda	2011–2012	263	160	39.8 (9.76) vs 39.8 (15.22)*	32.7% vs 41.2%	Primary: 54.8% vs 58.1%	NA	NA	100%	15 items	9.6 vs 21.1%
**HSCL Cut-off>44**												
Kagee et al, 2010 [60]	South Africa	NA	85	NS	32.8 (7.6)*	24.7%	Secondary: 10%; Tertiary: 90%	NA	NA	yes/no	25 items	52.9% (distress)

Abbreviations: ART: Antiretroviral Therapy; CI: Confidence Interval; IQR: InterQuartile Range; NA: Not available; SD: Standard Deviation.

*Mean (SD)

** Median (IQR)

NB: Articles are presented in function of ART status of included patients

**Table 7:** Severity of depressive disorders in HIV-infected adults evaluated by others scales.

First Author, year by chronological order	Country	Study years	HIV+ N	HIV- N	Demographic data			Clinical data			Tools	Main results or Prevalence (%) (95% CI)
					Age (years)	Men (%)	Education level (%)	CD4 cell count /mm^3^	CDC/WHO stage (%)	ART (%)		
**Hospital Anxiety and Depression Scale (HADS)**												
Pappin et al, 2012 [67]	South Africa	2007–2008	716	NA	36 ** (30.8–42.8)	24.3%	• Primary: 31% • Secondary: 45.8%	NA	NA	100%	Information available on depression only	25.4%
Wouters et al, 2012 [68]	South Africa	2007–2008	716	NA	37.2 (8.9)*	24%	Secondary / post-secondary: 57.6%	NA	NA	100%	Information available on depression only	• Mild or subclinical 15.3% • Moderate 8.8% • Severe 1.3%
**Others than HADS**												
Do et al, 2010 [69]	Botswana	2005	300	NA	24-35yo 64%	23.7%	• Secondary: 55.7% • Tertiary: 17.7%	NA	NA	100%	Questions + European Quality of life (EQ-5D) + BDI	28.3%. Of them: 4.6% mild; 31.8% moderate; 21.2% severe
Gaynes et al, 2012 [37]	Cameroon	2010	400	NA	• 41 • (34–47)**	26%	Post-Primary (>6 years): 39%	NA	NA	100%	QIDS (n = 29)	• Moderate : 34.4% • Severe : 59% • Very severe : 7%
Berhe et al, 2013 [70]	Ethiopia	2011–2012	269	NA	• 35-44yo: 40.5%; • 45-54yo: 19%	48%	• Secondary/ 22.7% • Tertiary: 30.5%	NA	NA	100%	Hamilton Depression Rating Scale (HAM-D)	• Mild 35.69% • Moderate 8.18%
Bongongo et al, 2013 [71]	South Africa	NA	117	NA	• 36*; • 31-39yo: 41.9%	29.9%	NA	NA	NA	100%	Zung self-rating depression scale	• Mild 69.2% • Moderate 1.7% • Severe 0.9%
Negash et al, 2013 [72]	Ethiopia	NA	355	NA	36.4*;	36.6%	NA	NA	WHO 3/4: 16.1% / 3.9%	100%	Interview with section dedicated to depression evaluation	5.4%
Yeji et al, 2014 [73]	South Africa	2007–2008	272	NA	37 (8.6)*;	21.3%	• Primary: 28.3% • Secondary: 38.9%	CD4 ≤200 : 30.9%	NA	100%	12-item General Health Questionnaire (GHQ12)	33 to 38% depending on the method used
Karambé et al, 2010 [74]	Mali	2004–2005	286	NA	36*; Range 18–65	47.59%	No schooling: 56.01%	CD4<200: 89.7%	NA	48.8%	NA	45.8% (most dominant among other psychological disorders)
Ramirez-Avila et al, 2012 [75]	South Africa	2006–2008	1,545	NA	34 (28–41)**	50%	• Some high school: 45.2%; • >high school: 30.5%	CD4<200: 59% (n = 1,126)	NA	628 eligible. Among them: 40% started	5-item Mental Health Index (MHI-5)	55%
Wagner et al, 2012 [40]	Uganda	2010–2011	602	NA	NA	NA	NA	NA	NA	49.8%	NA	57%

Abbreviations: ART: Antiretroviral Therapy; CI: Confidence Interval; IQR: InterQuartile Range; NA: Not available; SD: Standard Deviation.

*Mean (SD)

** Median (IQR). NB: Articles are presented in function of ART status of included patients

#### CES-D

Results are presented in Table 3.

**Meta-analysis**

**Fig 3:**
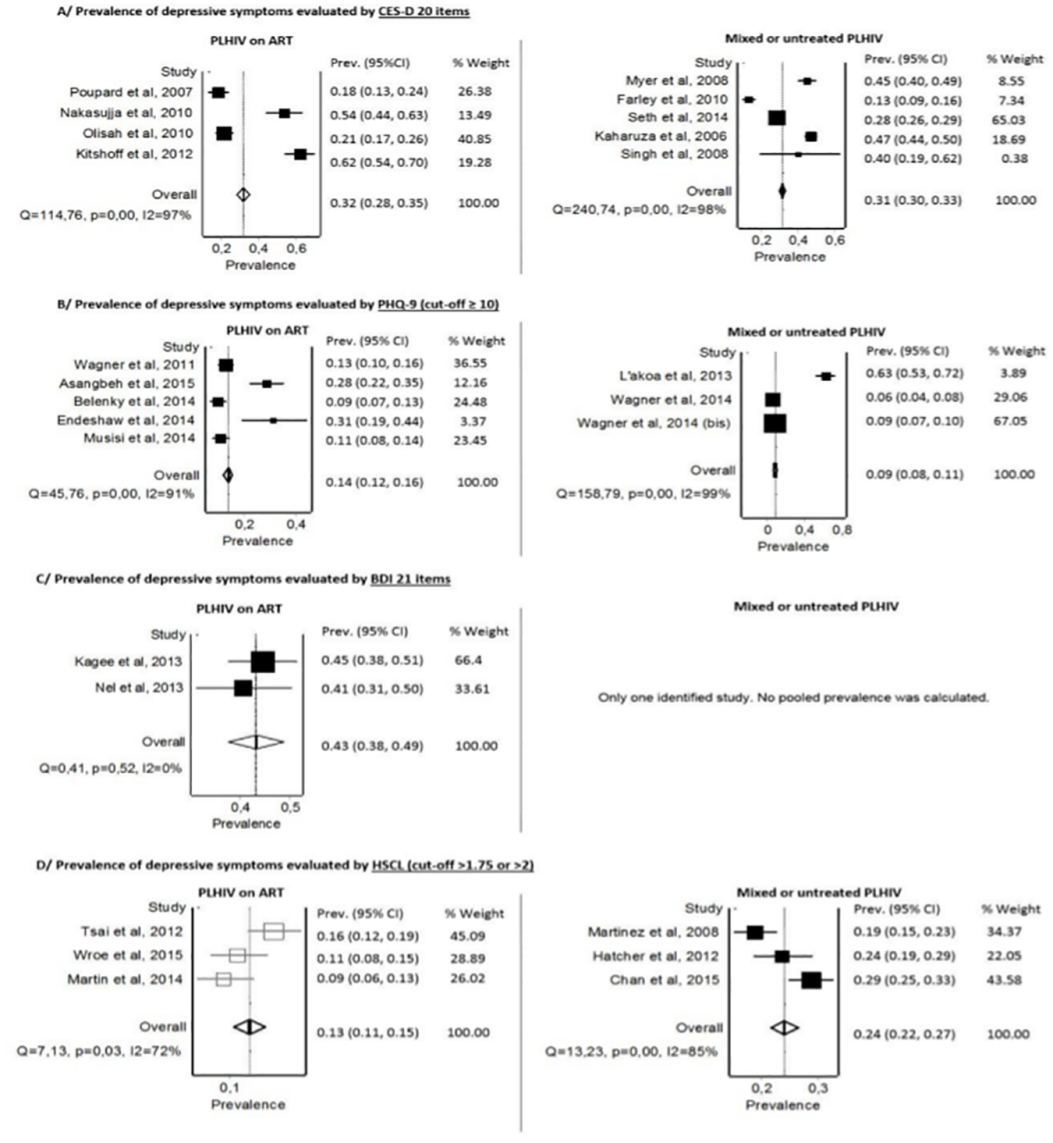
Forest plots presenting the prevalence of depressive symptoms according to the tool used and the treatment status. The between-study heterogeneity is reported using I^2^ statistics and its associated p-value. Prev: Prevalence; CI: confidence interval.

Based on the 20-item CES-D (cut-off≥16), the pooled prevalence of depressive symptoms among untreated or mixed groups of PLHIV was 31% [95% CI: 30–33] with a high between-group heterogeneity (I^2^ = 98%) (Fi 3A-right) [22,23,45–47]. Among the PLHIV on ART and based on the 20-item CES-D (cut-off≥16), the pooled prevalence of depressive symptoms was 32% [95% CI: 28–35] with a high between-group heterogeneity (I^2^ = 97%) (Fig 3A-left) [36,42–44]. In Poupard et al, different cut-off points were used for men (≥17) and women (≥23) [42].

**Qualitative review**

Among untreated or mixed groups of PLHIV, the CES-D scale without the somatic items, an approach limiting confounding of depression with HIV-related symptoms, was only used in one report with a prevalence of 30% [48]. Based on the 10-item CES-D, 40.9% PLHIV had higher-than-threshold levels of depressive symptoms [50]. Among the PLHIV on ART, it has been shown that after three and six months on ART, the prevalence decreased in one report and remained significantly higher in PLHIV initiating ART than among uninfected subjects at three months [43]. Based on the 10-item CES-D and among the PLHIV on ART, 8.7% of male and 13.1% of the women had higher-than-threshold levels of depressive symptoms [49].

#### PHQ-9

Results are presented in Table 4.

**Meta-analysis**

Based on the PHQ-9 (cut-off ≥10), the pooled prevalence of depressive symptoms among untreated or mixed groups of PLHIV was 9% [95% CI: 8–11] with a high between-group heterogeneity (I^2^ = 99%) (Fig 3B-right) [55–57]. Among the PLHIV on ART and based on the PHQ-9 (cut-off≥10), the pooled prevalence of depressive symptoms was 14% [95% CI: 12–16] with a high between-group heterogeneity (I^2^ = 91%) (Fig 3B-left) [51–54,76].

#### BDI

Results are presented in Table 5.

**Meta-analysis**

In untreated or mixed groups of PLHIV, no pooled prevalence was calculated considering the limited number of studies available. Based on the BDI (21 items), the pooled prevalence of depressive symptoms among PLHIV on ART was 43% [95% CI: 38–49] with no between-group heterogeneity (I^2^ = 0%) (Fig 3C-left) [9,59]. However, the cut-off scores were different from one study to another.

**Qualitative review**

In a series of PLHIV whose 97.5% were on ART, the prevalence figures of mild, moderate and severe depressive symptoms, based on fast screening BDI (7 items) were 59%, 27% and 14%, respectively [39]. In comparison to HIV-uninfected patients, ART-naïve PLHIV presented higher scores on BDI [77]. Using the 21-item BDI, prevalence figures of 24.7%, 20% and 17.6% of mild, moderate and severe depressive symptoms were respectively reported [60].

Among PLHIV on ART, in a longitudinal study using the BDI without somatic symptoms, the prevalence was relatively stable between baseline (55.8%) and after 3 months of follow-up (49.6%) [58].

#### HSCL

Results are presented in Table 6.

**Meta-analysis**

Based on the HSCL (cut-off>1.75 with 11–15 items) (Table 2D), the pooled prevalence of depressive symptoms among untreated or mixed groups of PLHIV was 24% [95% CI: 22–27] with a high between-group heterogeneity (I^2^ = 85%) (Fig 3D-right) [63–65]. Some studies specified that they used the scale without somatic symptoms [63,65]. Among the PLHIV on ART, the pooled prevalence of depressive symptoms was 13% [95% CI: 11–15] with a low between-group heterogeneity (I^2^ = 72%) (Fig 3D-left) [8,61,66].

**Qualitative review**

Among untreated or mixed groups of PLHIV, using 25 items and a cut-off of 44, one study reported a prevalence of 52.9% [60].

In PLHIV on ART living in Uganda, an unexpected lower prevalence (9.6%) was reported in comparison to uninfected subjects (21.1%) [66]. The authors explained this discrepancy by possible false negative observations in the general population (since the HIV prevalence in the study area was low) and by positive effects of counseling and ART in PLHIV. Using HSCL-25 items, one study reported a prevalence of 38% in treated PLHIV [62].

#### Other scales

Results are presented in Table 7.

**Meta-analysis**

No pooled prevalence was calculated due to the variety of scales used.

**Qualitative review**

Based on depression screening scales which are infrequently used, the prevalence of depressive symptoms ranged between 45.8% and 57% [40,74,75] in mixed groups of PLHIV whereas in PLHIV on ART, the prevalence varied between 2.6% and 66% [37,67–73].

### Associated factors

For MDD, we present the associated factors identified with the MINI only. For the severity of depressive symptoms, we present the associated factors identified with the two most used scales, i.e. CES-D 20 items and PHQ-9 and with the HSCL which presents the less heterogeneity. In each instance, the results are broken down according to the ART status.

#### Factors associated with MDD

In untreated or mixed groups of PLHIV, while one study reported no link between low CD4 count and depression [78], an association between low CD4 count and MDD was observed [31,32]. MDD was also associated with disability [31], being of younger age [32], being female [79], stigma [32], opportunistic infections [32], unemployment [6] and negative life events [31,79]. No association was reported with cognitive impairment [33], and educational level [32].

In PLHIV on ART, MDD was associated with WHO clinical stage 3 or 4 [29], poor health-related quality of life [28], additional comorbidities such as tuberculosis [29], prior history of MDD or other psychiatric disorders, such as past manic episode [29]. No association was reported with CD4 count, cognitive impairment, age and gender [29].

#### Factors associated with depressive symptoms

Among ART-naïve PLHIV, more severe depressive symptoms were observed in comparison to PLHIV on ART [45,56,57,66], except in one study using HSCL [63]. An improvement of the severity of depressive symptoms, based on HSCL, has also been observed over seven years of follow-up after initiating ART but only in women [65].

In untreated or mixed groups of PLHIV, even when the screening scale was restricted to somatic items, low CD4 count was the strongest predictor of severe depressive symptoms [47,55]. The severity of depression symptoms was also associated with poorer physical health [46,65], being female [46,47,65] (except in one report [55]) and being less educated [45,47]. The link between age and depression remained unclear [46,47]. Also, because of contradictory results, no consensus could be reached concerning substance abuse [46,55,63]. An association was reported with poor economic status [47], low social support [46] and negative life events [46] whereas no association was observed with an history of prior opportunistic infections [63]. These results were reported in only one publication each time.

In PLHIV on ART, the severity of depressive symptoms seemed unrelated to CD4 level [43,44], except in one [76]; whereas in another publication [52] a negative relationship was observed. Severity of depressive symptoms could be related to age [76] or low level of education [44] whereas no effect of gender was observed [42,53,76]. PLHIV with no more than primary school education seemed to have nearly a 2-fold risk of having more depressive symptoms [44]. Stigma [53], unemployment and lower income [44,76] seemed to be associated with severe depressive symptoms. Unemployed PLHIV could have a 3-fold risk to present severe depressive symptoms according to the same study [44]. No consensus could be reached concerning disease progression [43,76] whereas no association was observed for cognitive impairment [43].

### Quality of evidence

Based on GRADEpro, the overall quality of the studies selected for the meta-analysis was low, since all of them were observational (Tables B and C in S2 File). For the studies evaluating MDD or the severity of depressive symptoms, the quality of the studies for the different outcomes had to be downgraded to very low due to high inconsistency in study results, variability in study population (sample size, clinical information on patients) and small number of studies to calculate the pooled prevalence.

## Discussion

Since 2010, the number of published studies on depression in African PLHIV has substantially increased, highlighting the increasing concern on this emerging public health issue. Based on pooled prevalence estimates, the prevalence of MDD was 13% in PLHIV on ART and 24% in mixed/untreated groups of PLHIV. The prevalence of depressive symptoms varied between 14% and 32% in PLHIV on ART and between 9% and 31% in mixed/untreated groups of PLHIV according to the scale used. Overall, the pooled prevalence varied substantially according to the measurement scale used and also for a given scale even when the same cut-off or the same number of items was used in all studies. Few information on the procedures used to administer the scales (items’ number, cut-off, inclusion of somatic symptoms or not, etc.) were available, explaining at least a part of the variability in the data.

In clinical practice, depression is still usually under-diagnosed in SSA [3,70]. Depression is often considered as a logical outcome in PLHIV and is difficult to diagnose due to its common symptoms with HIV disease such as fatigue, sleeping problems, loss of appetite and cognitive impairment [6,39]. PLHIV report also more easily somatic symptoms than emotional or affective ones [38]. The lack of time available in primary health care settings, the lack of knowledge of the clinicians on mental health and the poor integration of mental health services in SSA contribute to the difficulties to identify patients with depression [80]. The scarcity of resources for mental health in low-income countries constitute barriers to take care adequately of those health issues [81]. A WHO report also highlighted these aspects in details [82]. A recent editorial in AIDS insisted on the need to act and to manage depression based on mhGAP guidelines (WHO 2010) [3]. The lack of validated and culturally-sensitive scales in SSA is also a limitation [3,83]. Most of the current depression screening tools also required extensive staff training and time to be administered.

The impact of ART on MDD or depressive symptoms makes no consensus so far in SSA studies. In a previous meta-analysis analyzing data from seven SSA countries, HIV treatment status had a significant effect on the prevalence of depressive symptoms but not on MDD [14]. Due to the limited amount of data available on ART-naïve or untreated PLHIV, we had to group publications of untreated or mixed groups of PLHIV, which could influence pooled prevalence estimates. Indeed, most of the publications focused on the established ART period.

Concerning other associated factors with MDD, quality of life, additional comorbidities, prior history of MDD might be associated factors in PLHIV on ART [28,29], whereas disability, being a woman, stigma and unemployment could be associated factors in mixed or untreated groups of PLHIV [31,32,79]. Concerning depressive symptoms, poor social conditions seem to be the main factor associated with depressive symptoms in PLHIV on ART [44,76] whereas in untreated or mixed groups of PLHIV, it seems to be immunosuppression and being female [46,47,65]. However, it is important to appreciate that we focused on papers using the most used scales to present the associated factors, leading to a limited number of publications. Consensus was difficult to reach when contradictory results were reported or when, for a given factor, only one study reported an association. Further studies might be conducted to explore this further and depict more precisely the differences according to ART status and changes occurring over time in these chronically ART-treated patients. The identification of other predictors could also allow the identification of at-risk PLHIV who could benefit to specific prevention or early care measures.

The chronic aspect of the disease status could play an important role in PLHIV-related depression as a stressor. Indeed, PLHIV are aware of having a disease under control but not cured with a need to take care of their health status to avoid complications. A recent review on chronic diseases has reported that depression is more common in chronic diseases like heart disease, stroke and cancer than in the general population [84](Clarke et al, 2009). Even if HIV infection was not included in that review, this work highlighted the fact the chronic status is major risk factor to develop depression. Further studies are needed in the PLHIV living in Africa to further depict this issue.

Because of the deleterious consequences of depression on PLHIV [3,7–11] and since depression is a modifiable condition, the new WHO recommendations for ART use highlight the need to screen and manage depression over the continuum of HIV care [85]. Studies on feasibility and effectiveness of culturally-sensitive psychotherapeutic intervention [86] or group-based counseling intervention [87] using a task-shifting approach have been recently conducted in SSA, leading to promising results and encouraging the necessity of identifying as early as possible PLHIV suffering from depression.

## Conclusion

The analysis of the available most recent literature confirmed that depression in PLHIV represents an increasing concern in SSA. The prevalence of depression is high even if the variability of the data does not allow to describe precisely the phenomenon and to identify strong predictors. Further studies are clearly needed to: 1) validate the most accurate tools according to the population of origin (language, education) as well as ART status; 2) provide practical guidelines for administration of the tests; and 3) evaluate MDD and/or depressive symptoms in most at-risk populations, including women, men having sex with men and older PLHIV. Depressed PLHIV are at increased risk of developing viral resistance and other poor outcomes because of low level of ART adherence, and more specifically older patients who have even a less efficient immune system. This may become a significant public health hazard, deserving preventive and corrective measures to assure PLHIV better quality of life and outcomes in SSA.

## Supporting information

S1 File Search strategy. (DOCX)

S2 File Supplementary tables. (DOC)
