# Supplementary material for: Prevalence and factors associated with depression in people living with HIV in sub-Saharan Africa: A systematic review and meta-analysis
Source: PLoS One. 2017 Aug 4;12(8):e0181960. doi: 10.1371/journal.pone.0181960 (PMC5544236; doi:10.1371/journal.pone.0181960)
Supplement: S1 File — (DOCX) [file pone.0181960.s001.docx]

**Search strategy for two databases**

**Research strategy in Pubmed:**

("HIV Infections"[Mesh] AND ("Depressive Disorder"[Mesh] OR "Depression"[Mesh])) AND ("Africa"[Mesh] OR "Africa South of the Sahara"[Mesh])

+ filter : from 1996/01/01 to 2016/04/30

+ filter: adults +19 years

**Research strategy in Scopus:**

( TITLE-ABS-KEY ( hiv )  AND  TITLE-ABS-KEY ( depression )  OR  TITLE-ABS-KEY ( depressive  disorders )  AND  TITLE-ABS-KEY ( africa )  OR  TITLE-ABS-KEY ( sub-saharan  africa )  AND  TITLE-ABS-KEY ( adults ) )

+ filter 1996 to present
